# COSMC Is Overexpressed in Proliferating Infantile Hemangioma and Enhances Endothelial Cell Growth via VEGFR2

**DOI:** 10.1371/journal.pone.0056211

**Published:** 2013-02-12

**Authors:** Jian-Jr Lee, Chia-Hua Chen, Ya-Hsin Chen, Miao-Juei Huang, John Huang, Ji-Shiang Hung, Ming-Ting Chen, Min-Chuan Huang

**Affiliations:** 1 Graduate Institute of Anatomy and Cell Biology, National Taiwan University College of Medicine, Taipei, Taiwan; 2 Research Center for Developmental Biology and Regenerative Medicine, National Taiwan University, Taipei, Taiwan; 3 Department of Surgery, National Taiwan University Hospital, Taiwan; 4 Division of Plastic Surgery, Department of Surgery, Cathay General Hospital, Taipei, Taiwan; University of Nebraska Medical Center, United States of America

## Abstract

Infantile hemangiomas are localized lesions comprised primarily of aberrant endothelial cells. COSMC plays a crucial role in blood vessel formation and is characterized as a molecular chaperone of T-synthase which catalyzes the synthesis of T antigen (Galβ1,3GalNAc). T antigen expression is associated with tumor malignancy in many cancers. However, roles of COSMC in infantile hemangioma are still unclear. In this study, immunohistochemistry showed that COSMC was upregulated in proliferating hemangiomas compared with involuted hemangiomas. Higher levels of T antigen expression were also observed in the proliferating hemangioma. Overexpression of COSMC significantly enhanced cell growth and phosphorylation of AKT and ERK in human umbilical vein endothelial cells (HUVECs). Conversely, knockdown of COSMC with siRNA inhibited endothelial cell growth. Mechanistic investigation showed that O-glycans were present on VEGFR2 and these structures were modulated by COSMC. Furthermore, VEGFR2 degradation was delayed by COSMC overexpression and facilitated by COSMC knockdown. We also showed that COSMC was able to regulate VEGF-triggered phosphorylation of VEGFR2. Our results suggest that COSMC is a novel regulator for VEGFR2 signaling in endothelial cells and dysregulation of COSMC expression may contribute to the pathogenesis of hemangioma.

## Introduction

Infantile hemangiomas are characterized by an aberrant growth of endothelial cells in the infant age. They are the most common tumors in infants and children. The incidence is ranged from 1.1 to 2.6%, with the highest estimates approaching 10 to 12% [1]. The life cycle of hemangiomas differs from that of most tumors in that it progresses from a phase of rapid proliferation followed by spontaneous involution [2]. During the proliferative phase, hemangiomas are composed of densely packed endothelial cells that form small capillaries [2]. While in the involuting phase, the capillaries have larger lumens with thickened capillary basement membranes, less-plump endothelial cells, and fibrofatty replacements of the lesion [3].

Angiogenesis is crucial for blood vessel formation and embryonic development. Vascular endothelial growth factor (VEGF) is the most critical growth factor expressed at the sites of angiogenesis and its expression levels are closely correlate with the spatial and temporal events of blood vessel development [4]. VEGF receptor 2 (VEGFR2, KDR/Flk-1) is the receptor that mediates endothelial cell responses to VEGF. Interestingly, VEGFR2-dependent signaling has been found to be upregulated in hemangioma endothelial cells [5], however, the mechanisms accounting for the increased VEGFR2 activity in hemangiomas remain unclear.

Glycosylation is the most common post-translational modification of proteins. Changes in carbohydrates, such as Tn and T antigens, are often correlated with tumor progression and prognosis in human cancers [6], [7]. Two major types of protein glycosylation in mammalian cells exist: N-linked and O-linked. The most frequently occurring *O-*glycosylation is the mucin-type, initiated by the transfer of *N*-acetylgalactosamine (GalNAc) to serine or threonine residues [8]. Tn antigen has a relatively simple structure composed of GalNAc with a glycosidic linkage to serine/threonine residues on glycoproteins (GalNAc-Ser/Thr). The Tn antigen is further modified to become T antigen (Galbeta1,3GalNAc) by core 1 ß1,3-galactosyltransferase (C1GALT1 or T-synthase) in the Golgi apparatus of cells [9]. Expression of an active T-synthase is uniquely dependent on the molecular chaperone COSMC [10].

Mice deficient in T synthase or COSMC are both embryonically lethal [11], [12]. Mosaic mice with dominant deletion of COSMC develop hemorrhages in the lung and GI tract, bloody chylous ascites, and growth retardation, which resembles the observations in mice with a conditional deletion of T-synthase in endothelial and hematopoietic cells [12]. These findings suggest that deficiency in endothelial cell O-glycans leads to blood/lymphatic misconnections and that T synthase and its chaperone COSMC are required for correct development of blood vessels [13]. Although several lines of evidence suggest critical roles of COSMC in endothelial cells the expression and functions of COSMC in hemangiomas are still unknown.

In the present study, we showed that COSMC and T antigen were overexpressed in human proliferating hemangiomas. We also demonstrated that COSMC overexpression increased cell growth in HUVECs and PI3K inhibitor, LY294002 significantly blocked the enhanced cell growth. Our results are the first to show that O-glycans are present on VEGFR2. Furthermore, COSMC overexpression modulated O-glycosylation of VEGFR2 and increased VEGF-triggered phosphorylation of VEGFR2. Conversely, COSMC knockdown inhibited endothelial cell growth and suppressed VEGF-mediated signaling. These findings suggest that COSMC is a novel regulator for VEGFR2 signaling and endothelial cell proliferation, which provides new insights into the mechanism of hemangioma development.

## Materials and Methods

### Clinical tissue collection

Human hemangioma tissues were obtained from the Department of Surgery, Cathay General Hospital, Taipei, Taiwan. The local hospital ethics committee approved the use of human tissues for this study, and written consent was obtained from patients before the collection of samples. For immunohistochemistry, specimens were fixed in 4% (w/v) paraformaldehyde/PBS at 4°C overnight. For RNA extraction, specimens were soaked in RNA*later* (Qiagen, Tokyo, Japan) at 4°C overnight and then stored at −20°C.

### Immunohistochemistry

Human hemangioma tissue sections were deparaffinized in xylene and rehydrated in a series of graded alcohols. After quenching the activity of endogenous peroxidase with 1% H_2_O_2_ in phosphate-buffered saline (PBS) for 10 min, the sections were rinsed three times with PBS and then incubated with 5% non-fat milk/PBS for 30 min to reduce non-specific bindings. Sections were incubated with an anti-COSMC polyclonal antibody (1∶30, Sigma, St. Louis, MO), biotinylated peanut agglutinin (PNA) (1∶250, Vector Laboratories, Burlingame, CA), or biotinylated *Vicia villosa* agglutinin (VVA) (1∶500, Vector Laboratories, Burlingame, CA) diluted with 1% bovine serum albumin (BSA)/PBS for 16 h at 4°C. After rinsing twice with PBS, Super Sensitive™ Link-Label immunohistochemistry Detection System (BioGenex, San Ramon, CA) was used and the specific immunostaining was visualized with 3,3-diaminobenzidine liquid substrate system (Sigma, St. Louis, MO). All sections were counterstained with hematoxylin for 1 min and mounted with UltraKitt (Mallinckrodt Baker, Inc., Phillipsburg, NJ). Negative controls were performed by replacing the primary antibody with a control IgG at the same concentration.

### Real-time RT-PCR

Total cellular RNA was isolated from hemangioma tissues or cells grown to 70% confluence by use of the Trizol reagent (Invitrogen, Carlsbad, CA) according to manufacturer protocols as previously described [14]. For cDNA synthesis, 2 µg of total RNA were used as templates in a 25 µl reverse transcription reaction. For *GAPDH* detection, sense and anti-sense primers were 5′-ACAGTCAGCCGCATCTTCTT -3′ and 5′- GACAAGCTTCCCGTTCTCAG -3′, respectively. For detection of *COSMC*, sense and anti-sense primers were 5′-TTTGAAGGGTGTGATGCTTG -3′ and 5′- ATGCGCTCATCCTCTGAAAT -3′, respectively. PCR products were separated by 1% agarose gels. For real-time PCR reactions, quantitative PCR System Mx3000P (Stratagene, La Jolla, CA) was used according to manufacturer's protocol. Briefly, reaction was performed in a 25-µl volume with 2 µl cDNA, 400 nM each of sense and anti-sense primers, and 12.5 µl Brilliant_R_SYBR_R_Green QPCR Master Mix (Stratagene, La Jolla, CA). PCRs were incubated for 15 min at 95°C followed by 40 amplification cycles with 30-s denaturation at 95°C, 50-s annealing at 54°C, 30-s extension at 72°C. Samples were analyzed in triplicate, and product purity was checked through dissociation curves at the end of real-time PCR cycles. Relative quantity of gene expression normalized to *β-actin* was analyzed with MxPro Software (Stratagene, La Jolla, CA).

Human umbilical vein endothelial cells (HUVECs) (Lonza, Walkersville, MD) were cultured in Clonetics® EGM-2 BulletKit (Lonza, Walkersville, MD) according to the manufacturer's instruction. Human endothelial cell line EA.hy926 was a gift from Drs. Shu-Huei Wang and Hsiu-Ni Kung (Graduate Institute of Anatomy and Cell Biology, National Taiwan University College of Medicine, Taiwan) and was maintained in Dulbecco's modified Eagle's medium (DMEM; Thermo scientific, Barrington IL) containing 10% FBS, 100 IU/mL penicillin, and 100 µg/mL streptomycin (Invitrogen, Carlsbad, CA) in a humidified tissue culture incubator at 37°C and 5% CO_2_ atmosphere. COSMC/pcDNA3.1 [15] (a gift from Dr. Richard D. Cummings at the Emory University School of Medicine, Atlanta, USA) and control pcDNA3.1 (Mock) (Invitrogen, Carlsbad, CA) were transfected into HUVECs (3-5 passages) using an Amaxa Nucleofector™ (Lonza, Walkersville, MD) and the HUVEC Nucleofector kit (Lonza, Walkersville, MD) according to the manufacturer's instructions. For COSMC knockdown, duplex siRNA against *COSMC* and non-targeting control siRNA were purchased from Invitrogen. Cells were transfected with siRNAs using Lipofectamine RNAiMAX (Invitrogen, Carlsbad, CA) at a final concentration of 10 nM siRNA according to the manufacturer's instruction. After 48 h of transfection, cells were used for experiments. For analysis of cell signaling, cells were starved for 4 h and then treated with 10% FBS or 20 ng/mL of VEGF (Sigma, St. Louis, MO).

### Western blot analysis

Equal amounts of cell or tissue lysates were electrophoresed on an SDS-PAGE and transferred to a nitrocellulose membrane. Membranes were incubated with anti-COSMC polyclonal antibody, anti-β-actin monoclonal antibody (Sigma, St. Louis, MO), anti-phospho-ERK monoclonal antibody, anti-ERK1/2 polyclonal antibody, anti-phospho-AKT, anti-VEGFR2 monoclonal antibody, anti-phospho-Tyr1175 VEGFR2 monoclonal antibody (Cell Signaling Technology, Beverly, MA), or biotinylated PNA (Vector Laboratories, Burlingame, CA). The membrane was incubated with HRP-conjugated secondary antibodies (Santa Cruz Biotechnology, Santa Cruz, CA). Signals were visualized with ECL reagents (Amersham Biosciences, Buckinghamshire, UK) and quantified with ImageQuant 5.1 (Amersham Biosciences, Buckinghamshire, UK).

### Trypan blue exclusion assay

Cells (4×10^4^) were seeded in 6-well plates with RPMI 1640 containing 10% FBS (PAA Laboratories, New Bedford, MA). Viable cells in triplicate wells were determined at 24 h intervals for 72 h using hemocytometer with trypan blue exclusion staining (Sigma, St. Louis, MO).

### MTT cell proliferation assay

For viability and proliferation analysis, cells were seeded at 3×10^3^ per well in 96-well plates. Triplicates of each cell group were plated. The 3-(4,5-Dimethylthiazol-2-yl)-2,5-diphenyltetrazolium bromide (MTT) reagents (Sigma, St. Louis, MO) were added to each well, and absorbance was measured according to the manufacturer's instructions.

### Lectin pull down assay

For lectin pull down assays, 200 µg of cell or tissue lysates were incubated with PNA or VVA agarose beads (Vector Laboratories, Burlingame, CA) overnight at 4°C. The pulled down proteins were then subjected to Western blotting.

### Chemical inhibition

The MEK inhibitor PD98059 and PI3K inhibitor LY294002 (Calbiochem, San Diego, CA), and VEGF receptor tyrosine kinase inhibitor SU1498 (Santa Cruz Biotechnology, Santa Cruz, CA) were dissolved in dimethyl sulfoxide (DMSO) to prepare a 10 mM of stock solution. For the inhibition of cell growth, 10 µM PD98059, 5 µM PY294002, or 10 µM SU1498 were used. DMSO was used for a solvent control.

### VEGFR2 degradation assays

Cells were cultured on 12-well plates and serum starved for 4 h. Cycloheximide (10 µg/ml) (Sigma, St. Louis, MO) and 20 ng/ml of VEGF were added to the cells for different time points. Cells were lysed in lysis buffer containing Tris 20 mM, pH 8.0, NaCl 137 mM, 1% NP-40, 10% glycerol, Na_3_VO_4_ 2 mM, β-glycerophosphate 2 mM, PMSF 2 mM, and 1% protease inhibitor cocktail (Sigma, St. Louis, MO). VEGFR2 protein levels were determined by Western blotting. β-actin was used as an internal control. The 0 h-control was set to 100% and the detected level of VEGFR2 was calculated as the percentage of 0 h-control for each time point.

### Statistical analyses

Student's *t*-test was used for statistical analyses. Data are presented as means ± S.D. Chi-square tests were used to test associations between COSMC and T antigen expression. *P*<0.05 was considered significant.

## Results

### COSMC is overexpressed in proliferating hemangiomas

**Figure 1:**
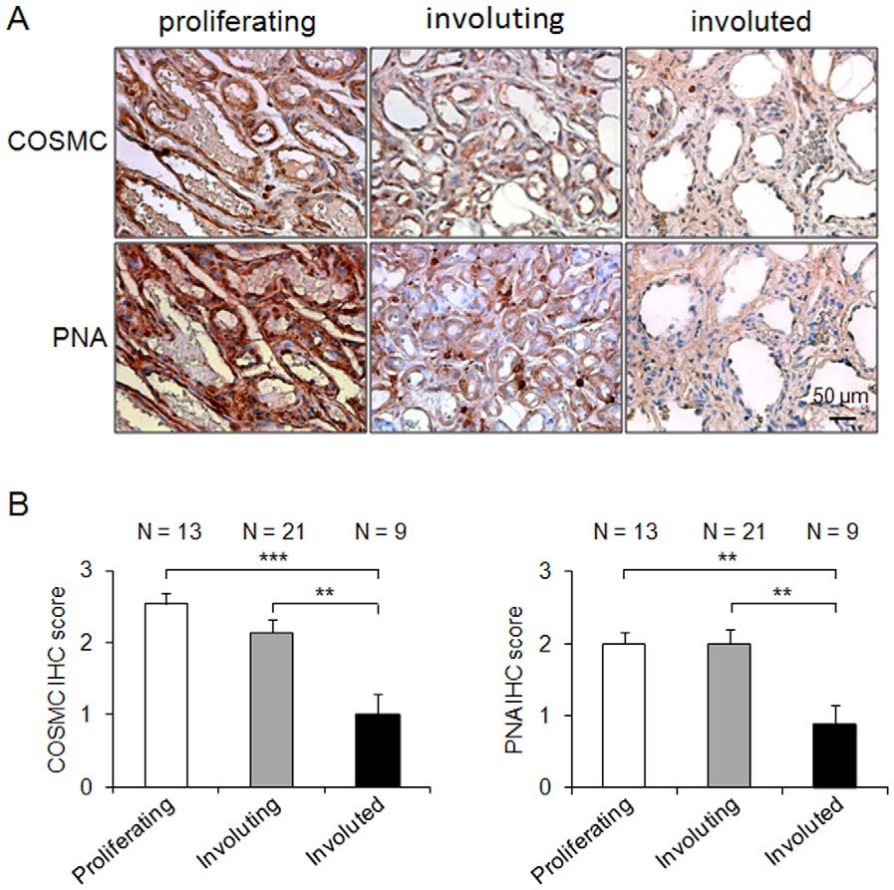
COSMC is overexpressed in proliferating hemangiomas. (A) Immunohistochemistry of human hemangioma tissues. Paraffin-embedded hemangiomas in proliferating (N = 13), involuting (N = 21), and involuted (N = 9) phases were immunostained with anti-COSMC antibody or biotin-conjugated peanut agglutinin (PNA). Representative images are shown. Scale bars: 50 µm. Negative controls did not show specific staining (data not shown). (B) COSMC is overexpressed in proliferating and involuting hemangiomas compared with involuted hemangiomas. Intensity of immunostaining was quantified. N = patient numbers. Data are presented as means ± SEM. **P<0.01; ***P<0.001.

To investigate the expression of COSMC in hemangiomas, immunohistochemistry was performed. Our results showed that COSMC expression level was the highest in proliferating hemangiomas, followed by involuting hemangiomas, and the lowest in involuted hemangiomas (Fig. 1). We also observed that COSMC expression in the surrounding normal blood vessels was not detectable or relatively weak, similar to that found in involuted hemangiomas (Fig. S1)(if accepted, production will need this reference to link the reader to the figure). These findings suggest that COSMC is overexpressed in proliferating hemangiomas compared with involuted hemangiomas which entails that COSMC may play a role in hemangioma development.

Since COSMC is a chaperone for T synthase to synthesize T antigen which is significant in tumor malignancy [16], we analyzed whether T antigen was also overexpressed in proliferating hemangiomas. Immunohistochemical analyses with PNA indicated that T antigen was also overexpressed in proliferating hemangiomas compared with involuted hemangiomas (Fig. 1). Furthermore, our data showed that proliferating hemangiomas expressed Tn antigen, as revealed by *Vicia villosa* agglutinin (VVA) staining (Fig. S2) (if accepted, production will need this reference to link the reader to the figure). The staining was augmented after neuraminidase treatment, indicating the presence of sialyl Tn structures in proliferating hemangiomas. These results suggest that both COSMC is overexpressed in the proliferating phase of infantile hemangiomas compared with the involuted phase, which is associated with increased T antigen expression.

### COSMC overexpression enhances T synthase and surface T antigen expression in HUVECs

**Figure 2:**
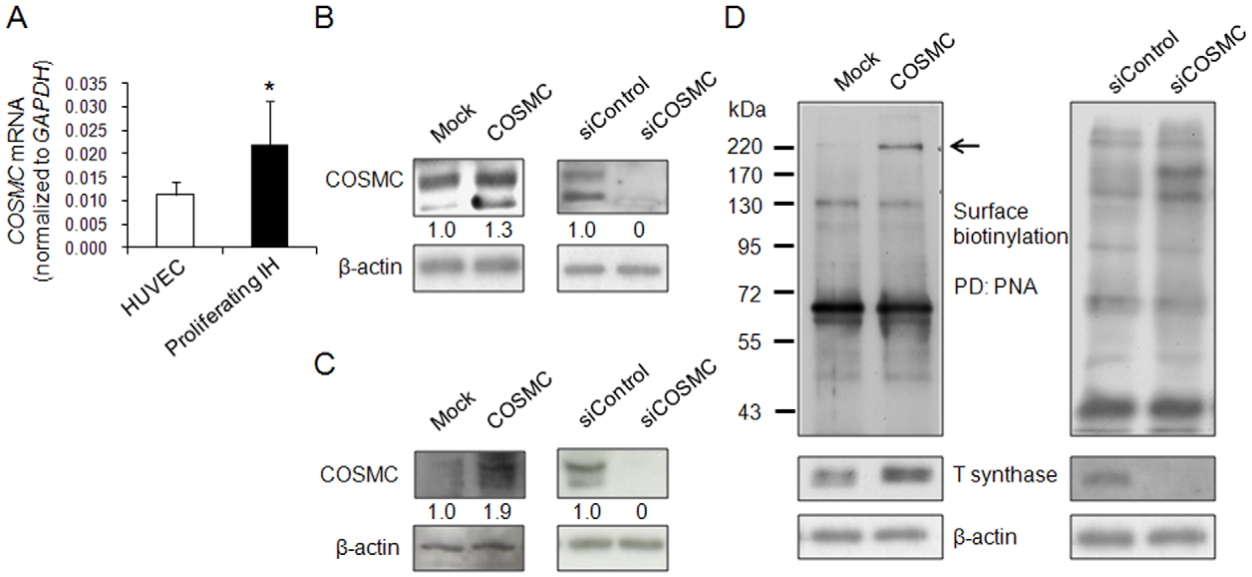
Expression of COMSC in HUVECs and EA.hy926 cells. (A) Proliferating infantile hemangiomas (IHs) (n = 3 patients) express higher levels of COSMC than HUVECs (n = 3 batches). *COSMC* mRNA levels were analyzed by real-time RT-PCR. *P<0.05. In lower panel, *COSMC* is overexpressed in HUVECs. (B) Western blots showing COSMC overexpression (left panel) and knockdown (right panel) in HUVECs. β-actin is a loading control. Relative intensity of signals on Western blots was quantified by ImageQuant5.1. (C) COSMC overexpression and knockdown in human endothelial cell line EA.hy926. Relative intensity of signals on Western blots was quantified by ImageQuant5.1. (D) COSMC overexpression and knockdown modulate cell surface carbohydrates on HUVECs. Left panel, COSMC overexpression enhances T synthase and T antigen expression in HUVECs. HUVECs were cell surface biotinylated, lysed, pulled down (PD) with PNA, and then blotted with streptavidin-HRP. The arrow indicated that a protein band with molecular mass of 220-kDa has increased PNA binding in COSMC-transfected HUVECs. Cell lysates were Western blotted for detecting T synthase expression and β-actin was used as a loading control. Right panel, COSMC knockdown increased glycoproteins pulled down by VVA, which recognizes Tn antigen.

To investigate the roles of COSMC in endothelial cells, we first analyzed COSMC expression in HUVECs and proliferating hemangiomas. Real-time RT-PCR showed that HUVECs expressed lower levels of COSMC compared with proliferating infantile hemangiomas (Fig. 2A). COSMC overexpression or knockdown in HUVECs (Fig. 2B) and human endothelial cell line EA.hy926 (Fig. 2C) were achieved by transfection with *COSMC*/pcDNA3.1 or COSMC-specific siRNA. . We found that COSMC overexpression enhanced T synthase expression at protein levels, which in turn increased cell surface T antigen expression as reflected by an increased PNA binding (Fig. 2D, left panel). In contrast, COSMC knockdown decreased T synthase expression but increased Tn expression, as revealed by VVA binding (Fig. 2D, right panel). These findings suggest that COSMC can increase protein levels of T synthase and enhance T antigen expression in endothelial cells.

### COSMC overexpression enhances cell growth of HUVECs

**Figure 3:**
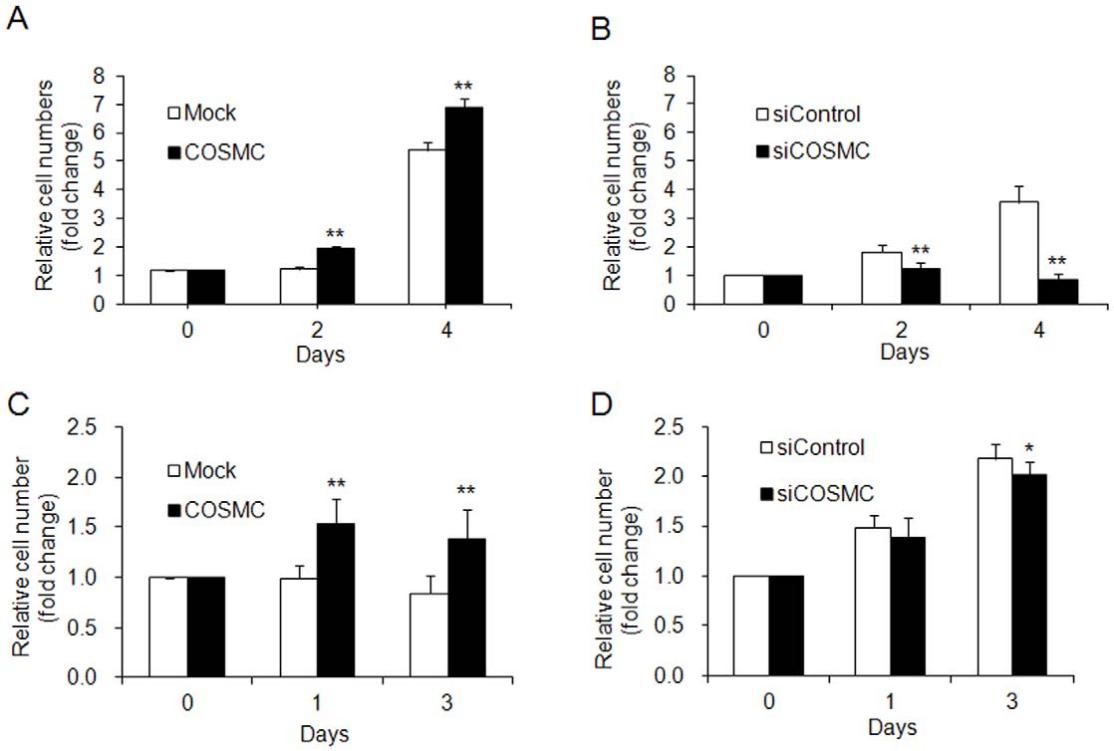
COSMC overexpression enhances cell growth in HUVECs. (A) Cell growth of HUVECs transfected with pcDNA3.1 control plasmid (open bars) or COSMC/pcDNA3.1 (closed bars) analyzed by trypan blue exclusion assays. (B) Cell growth of HUVECs transfected with control siRNA (open bars) or COSMC siRNA (closed bars) analyzed by trypan blue exclusion assays. (C) Cell growth of EA.hy926 cells overexpressing COSMC. (D) Cell growth of EA.hy926 cells with COSMC knockdown. Results are presented as means ± SD from three independent experiments. *P<0.05 and **P<0.01, compared with mock.

Since increased cell proliferation is a prominent phenotype for proliferating hemangiomas, we analyzed the effects of COSMC on cell proliferation in endothelial cells. Our results showed that overexpression of COSMC significantly enhanced cell proliferation in HUVECs by trypan blue exclusion assay (Fig. 3A). Although the increased levels of cell growth were dependent on passage times of HUVECs we consistently found that COSMC significantly increased cell growth at day 2 after transfection. Furthermore, knockdown of COSMC suppressed cell growth of HUVECs (Fig. 3B). Similar results were obtained by MTT assays (Fig. S3) (if accepted, production will need this reference to link the reader to the figure). To confirm the role of COSMC in cell growth of endothelial cells similar assays were performed in EA.hy926 cells. Comparable results were obtained in that COSMC overexpression enhanced cell growth (Fig. 3C), whereas COSMC knockdown suppressed cell growth (Fig. 3D). These results suggest that COSMC plays a role in regulating endothelial cell proliferation.

### COSMC overexpression modulates O-glycans on VEGFR2 and enhances phosphorylation of VEGFR2 in HUVECs

**Figure 4:**
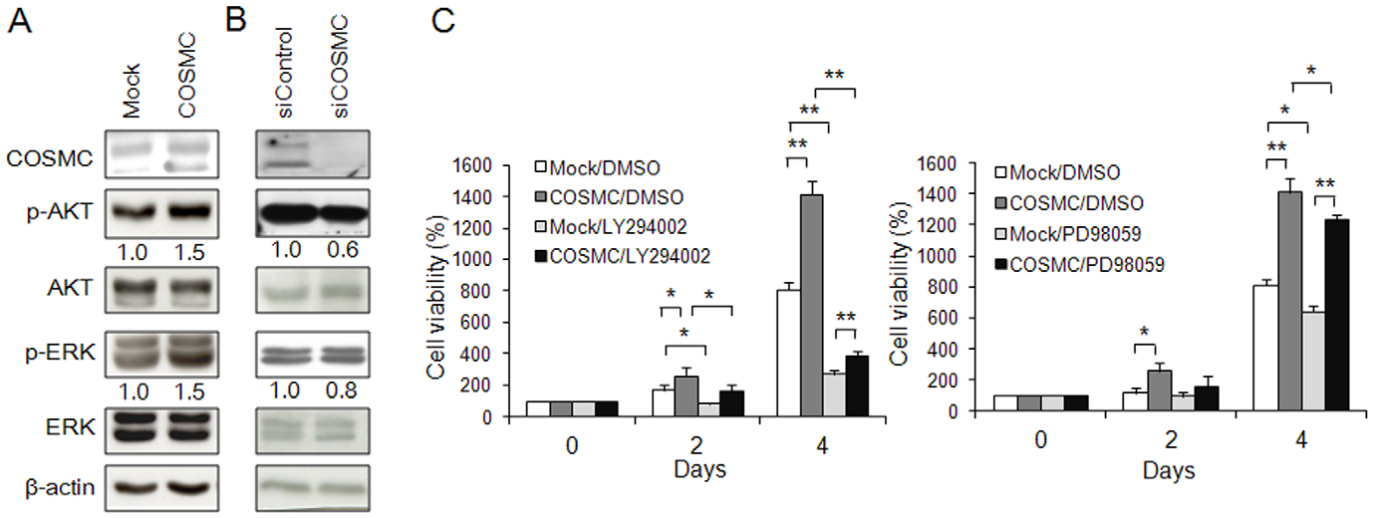
Roles of AKT and ERK signaling pathways in COSMC-enhanced cell proliferation. (A) COSMC overexpression enhances phosphorylation of AKT and ERK. Western blotting was performed to analyze protein expression. Representative images are presented. Relative intensity of signals was quantified by ImageQuant5.1 and shown. (B) COSMC knockdown inhibits phosphorylation of AKT and ERK. (C) Effects of AKT and ERK inhibitors on cell proliferation. HUVECs transfected with mock or COSMC plasmids were treated with DMSO control, 5 µM of LY294002, or 10 µM of PD98059. Cell viability was analyzed by MTT assays at different time points. Results are presented as means ± SD from three independent experiments. *P<0.05; **P<0.01.

Since AKT and ERK are two major signaling pathways known to regulate HUVEC proliferation we analyzed whether they were modulated by COSMC. HUVECs transfected with COSMC/pcDNA3.1 or COSMC siRNA, and their controls were serum starved for 4 h and then treated with 10% FBS for 10 min. Our results showed that phosphorylation of both AKT and ERK were increased by COSMC overexpression (Fig. 4A). COSMC knockdown suppressed phosphorylation of AKT and ERK (Fig. 4B). To evaluate the role of AKT and ERK in cell proliferation, HUVECs were treated with chemical inhibitors LY294002 and PD98059, respectively. MTT assays showed that LY294002 dramatically inhibited COSMC-enhanced cell growth (Fig. 4C, left panel). PD98059 inhibited cell proliferation to a lesser extent (Fig. 4C, right panel). These results suggest that COSMC enhances phosphorylation of AKT and ERK and the PI3K-AKT signaling pathway plays a predominant role in HUVEC proliferation.

### VEGFR2 is identified as an O-glycosylated protein and its O-glycosylation can be modified by COSMC in HUVECs

**Figure 5:**
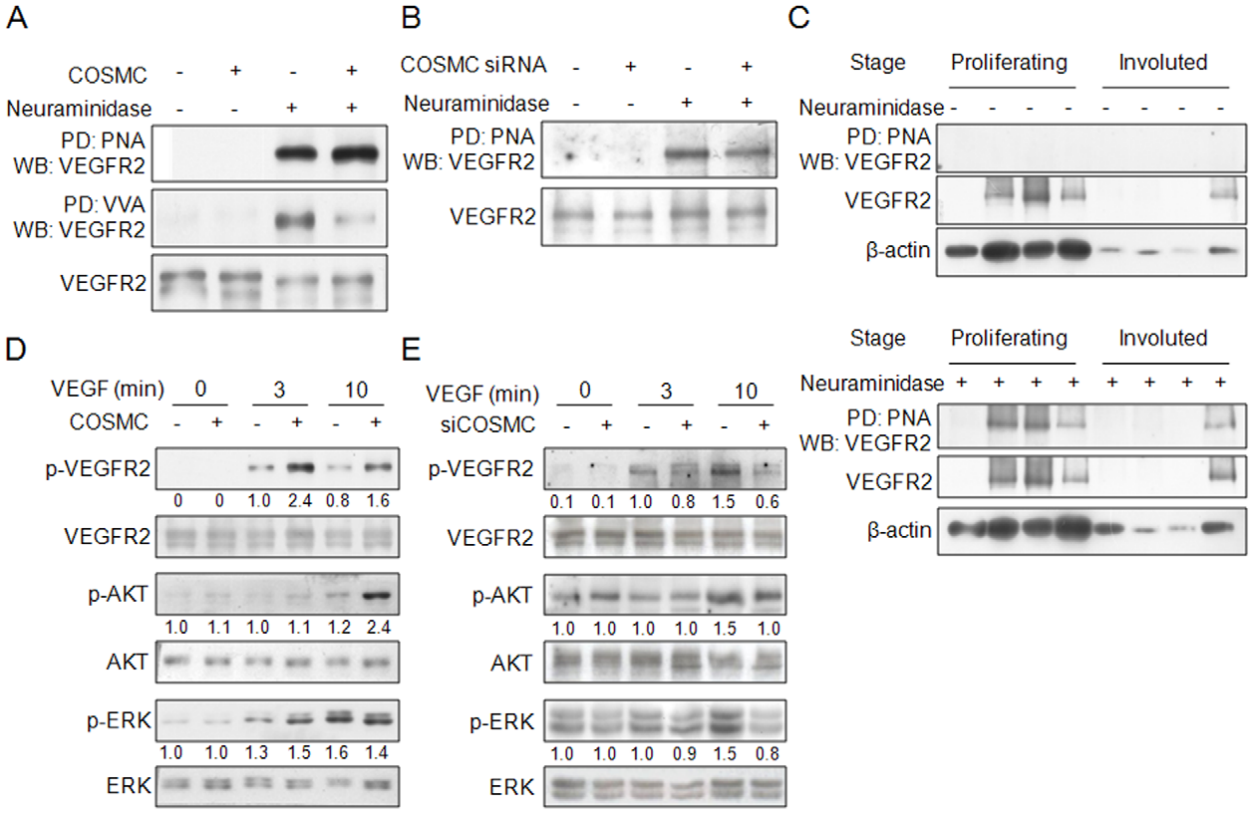
COSMC overexpression modulates O-glycans on VEGFR2. (A) Changes in O-glycans on VEGFR2 in COSMC overexpressing HUVECs. Cell lysates of HUVECs transfected with control or *COSMC* plasmids were treated with or without neuraminidase, pulled down with VVA or PNA lectins, then immunoblotted with anti-VEGFR2 antibody. β-actin is an internal control. (B) COSMC knockdown in HUVECs decreases binding of PNA to VEGFR2. (C) O-glycans are present on VEGFR2 in human primary hemangiomas. Tissue lysates of proliferating and involuted hemangiomas with (+) or without (−) neuraminidase treatment were pulled down (PD) by PNA and then immunoblotted with anti-VEGFR2 antibody. β-actin is an internal control. (D) COSMC overexpression enhances phosphorylation of VEGFR2 in HUVECs. HUVECs were serum starved for 4 h and then treated with 20 ng/ml of VEGF for different time periods. Phosphorylation of VEGFR2, AKT, and ERK were analyzed by Western blotting. β-actin is a loading control. Representative images from two independent experiments were shown. Signals on Western blots were quantified by ImageQuant5.1. (E) COSMC knockdown suppresses phosphorylation of VEGFR2 in HUVECs. Signals on Western blots were quantified by ImageQuant5.1.

To further investigate the molecular mechanism by which COSMC increased cell proliferation we attempted in identifying glycoproteins with increased T antigens by COSMC overexpression. Since VEGFR2 is a crucial endothelial growth factor receptor with a molecular mass around 220 kDa (data not shown) and a protein with increased T antigen was coincidentally found at 220 kDa (Fig. 2D, left panel) , we tested whether VEGFR2 was the mediator to enhance endothelial cell growth by COSMC overexpression. Consistent with previous observations, we found that VEGFR2 inhibitor SU1498 dramatically inhibited HUVEC growth (data not shown). Interestingly, our results showed that VEGFR2 could be easily pulled down by O-glycan-binding lectins VVA and PNA after neuraminidase treatment (Fig. 5A and 5B). Moreover, binding of PNA to VEGFR2 in COSMC-overexpressing HUVECs was increased, whereas binding of VVA to VEGFR2 was decreased (Fig. 5A). In contrast, knockdown of COSMC decreased PNA binding to VEGFR2 (Fig. 5B). In addition, we found that VEGFR2 in primary hemangioma tissues could be pulled down by PNA after removal of sialic acids (Fig. 5C) indicating that VEGFR2 carries O-glycans *in vivo*. These results suggest that VEGFR2 is O-glycosylated and its O-glycosylation is modulated by COSMC in HUVECs.

We next investigated whether COMSC could regulate VEGFR2 and its downstream signaling. Western blotting showed that VEGF-triggered phosphorylation of VEGFR2 was increased in COSMC-overexpressing HUVECs (Fig. 5D). We also observed that phosphorylation of AKT and ERK was enhanced. In contrast, knockdown of COSMC inhibited VEGF-mediated signaling, as revealed by decreased phosphorylation of VEGFR2 and its downstream signaling (Fig. 5E). These findings suggest that COSMC overexpression can enhance VEGF-induced phosphorylation of VEGFR2.

### COSMC regulates VEGFR2 degradation in endothelial cells

**Figure 6:**
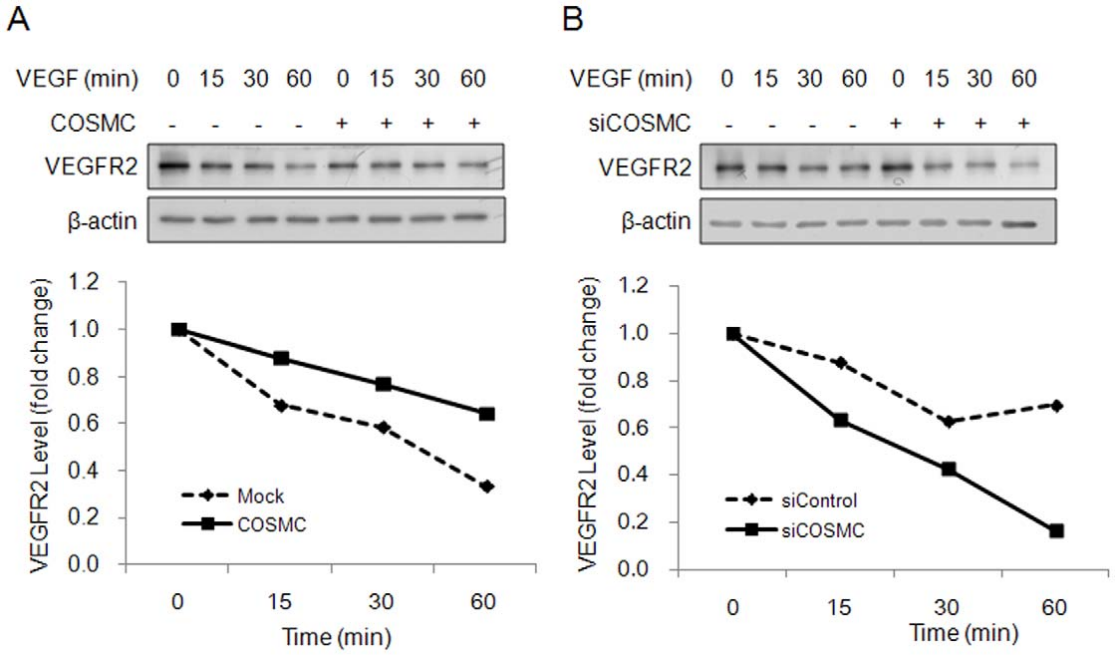
COSMC modulates protein degradation of VEGFR2. (A) COSMC overexpression delays degradation of VEGFR2. HUVECs were treated with cycloheximide (10 µg/ml) to block protein synthesis and 20 ng/ml of VEGF to trigger internalization and degradation of VEGFR2 for indicated time points. Upper panel shows representative Western blots. Lower panel shows signals on Western blots quantified by ImageQuant5.1 for HUVECs transfected with pcDNA3.1 control plasmid (dashed line) and UVECs transfected with COSMC/pcDNA3.1 (solid line). (B) COSMC knockdown facilitates degradation of VEGFR2. VEGFR2 degradation in HUVECs transfected with control siRNA (dashed line) or COSMC siRNA (solid line) was shown. Representative data from two independent experiments are presented.

To assess whether O-glycosylation could modulate VEGFR2 properties, we performed protein degradation assays in HUVECs. HUVECs were treated with cycloheximide to block protein synthesis and 20 ng/ml of VEGF was added at different time points to induce VEGFR2 internalization and degradation. Our results showed that VEGF was able to induce VEGFR2 protein degradation and COSMC overexpression delayed the degradation of VEGFR2 (Fig. 6A). Conversely, knockdown of COSMC facilitated degradation of VEGFR2 (Fig. 6B). These results suggest that COSMC can regulate VEGF-triggered degradation of VEGFR2.

## Discussion

Tn is the simplest mucin-type O-glycan, and could be further modified to generate sialyl Tn or T-related antigens, which are commonly found in tumorous tissues [7]. These tumor-associated carbohydrate antigens have been reported to be expressed in a variety of epithelial cancers, including breast, colon, lung, bladder, cervical, ovarian, stomach, and prostate, and they are used for development of cancer vaccines [7]. Expression of Tn/T-related carbohydrates often correlates with tumor progression and poor prognosis of diseases [16]–[18]. In consistent with previous reports, we found that T, Tn, and sialyl Tn were highly expressed in proliferating hemangiomas, whereas no or relatively low expression was observed in the endothelium of involuted hemangiomas or normal liver. The abundance of Tn and T carbohydrate structures may be of promising targets for developing diagnostic and therapeutic reagents for infantile hemangiomas.

In our study, we found that COSMC was overexpressed in proliferating hemangiomas compared with involuted hemangiomas. COSMC functions as a chaperone for folding and stabilizing T synthase, which is essential for its enzymatic activity to generate T antigen [15]. In addition, COSMC overexpression in HUVECs enhanced expression levels of T synthase protein, which in turn increased T antigen expression on cell surfaces. At molecular levels, we also demonstrated that COSMC overexpression increased sialyl T antigen expression on VEGFR2. Therefore, it is reasonable to conclude that COSMC exerts its biological functions mainly via increased protein levels of T synthase.

Our data suggested that the major O-glycans of VEGFR2 in HUVECs could be sialyl Tn and sialyl T since VEGFR2 could be easily pulled down by VVA and PNA after removal of sialic acids. Moreover, COSMC overexpression enhanced PNA binding, while VVA binding was decreased. We also found that PNA was able to bind to neuraminidase-treated VEGFR2 of primary hemangiomas, suggesting that VEGFR2 expresses sialyl T antigens *in vivo*. However, we did not observe enhanced sialyl T antigen expression in proliferating hemangiomas compared with involuted hemangiomas. One of the explanations could be that several factors, in addition to COSMC, collaboratively modulate the expression of T antigen on VEGFR2 *in vivo*. Interestingly, prediction of O-glycosylation sites using NetOGlyc 3.1 indicates that there are four potential O-glycosylation sites in the extracellular domain of VEGFR2. These results strongly suggest the presence of O-glycans on VEGFR2. To our knowledge, we are the first to report that VEGFR2 expresses O-glycans and changes in the O-glycans can modulate activity, protein stability, and signaling of VEGFR2. It will be of great interest to further investigate the exact structures and sites of O-glycans on VEGFR2 to understand how O-glycosylation modulates VEGFR2 activity.

VEGFR2 is an important surface receptor to regulate cellular properties of endothelial cells, including cell proliferation and angiogenesis [19]. The significance of VEGFR2 in infantile hemangioma cells has been documented, in which higher VEGFR2 activity, but not protein levels, has been suggested to be a key determinant for aberrant growth of hemangioma cells [5], [20], [21]. Mutations have been found in the kinase domain of VEGFR2, resulting in an increased kinase activity of VEGFR2 [5], [22]. However, these mutations are found only in rare cases of infantile hemangiomas. One of the other possibilities for the increased VEGFR2 activity may result from post-translational modification of VEGFR2. Our data showed that O-glycosylation modification of VEGFR2 could enhance VEGFR2 phosphorylation and increased its downstream signaling. Therefore, this study provides novel insights into the significant role of O-glycosylation in VEGFR2 activity and the molecular pathogenesis of infantile hemangiomas.

In this study, we showed that COSMC was overexpressed in proliferating hemangiomas, which was associated with increased T antigen expression. COSMC overexpression increased AKT and ERK1/2 signaling and enhanced endothelial cell growth in HUVECs. Mechanistic investigation showed that COSMC changed O-glycosylation and degradation of VEGFR2, and enhanced VEGF-mediated phosphorylation of VEGFR2 and its downstream signaling. These results indicate that the COSMC-enhanced endothelial growth is at least, in part, mediated through activation of VEGFR2. Since there are several potential acceptor substrates for T synthase in endothelial cells, it has to be noted here that other pathways may act in concert with VEGFR2 to regulate endothelial cell proliferation. In conclusion, our results suggest that COSMC is a novel regulator for VEGFR2 signaling in endothelial cells and may contribute to the aberrant growth of human infantile hemangioma. This study opens up avenues for treating VEGFR2-dependent diseases by targeting not only the receptors themselves but their glycosylation regulators.

## Supporting Information

Figure S1 **Immunohistochemistry of COSMC in normal blood vessels.** The arrows show relatively weak COSMC staining of the surrounding normal blood vessels in proliferating hemangioma, using tissue section obtained from the same tissue sample as used in Fig. 1A. Stars indicate sweat glands with strong COSMC staining. Scale bar, 50 µm.
(TIF)

Figure S2 **Immunohistochemistry of VVA in proliferating hemangioma and liver.** The proliferating infantile hemangioma, which is the same as in Fig. 1A, and human liver tissue with or without neuraminidase treatment were stained with VVA. Scale bar, 50 µm.
(TIF)

Figure S3 **Effects of COSMC on cell proliferation.** (A) COSMC overexpression increased cell proliferation of HUVEC cells. Cell proliferation was analyzed by MTT assays. (B) COSMC knockdown inhibited cell proliferation. Results are presented as means ± SD from three independent experiments. *P<0.05 and **P<0.01, compared with control.
(TIF)
