# Supplementary figures and images for: COSMC Is Overexpressed in Proliferating Infantile Hemangioma and Enhances Endothelial Cell Growth via VEGFR2
Source: PLoS One. 2013 Feb 12;8(2):e56211. doi: 10.1371/journal.pone.0056211 (PMC3570459; doi:10.1371/journal.pone.0056211)

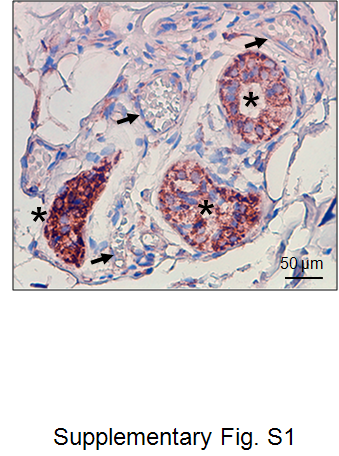

Supplement: Figure S1 — Immunohistochemistry of COSMC in normal blood vessels. The arrows show relatively weak COSMC staining of the surrounding normal blood vessels in proliferating hemangioma, using tissue section obtained from the same tissue sample as used in Fig. 1A. Stars indicate sweat glands with strong COSMC staining. Scale bar, 50 µm. (TIF) [file pone.0056211.s001.tif]

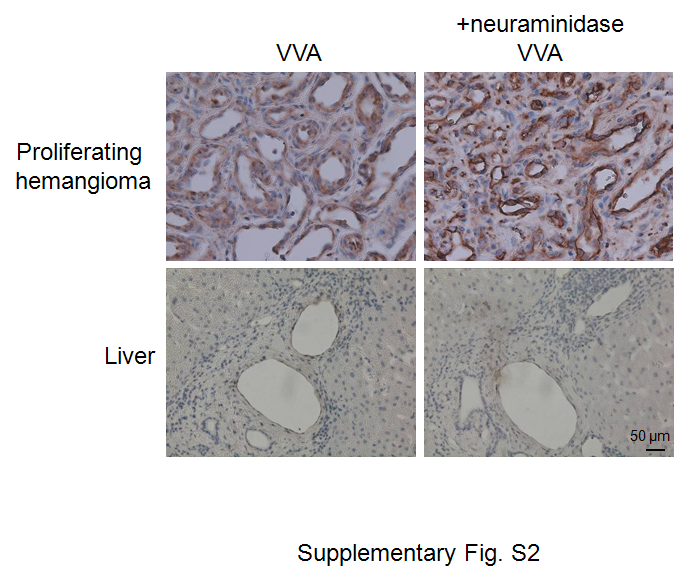

Supplement: Figure S2 — Immunohistochemistry of VVA in proliferating hemangioma and liver. The proliferating infantile hemangioma, which is the same as in Fig. 1A, and human liver tissue with or without neuraminidase treatment were stained with VVA. Scale bar, 50 µm. (TIF) [file pone.0056211.s002.tif]

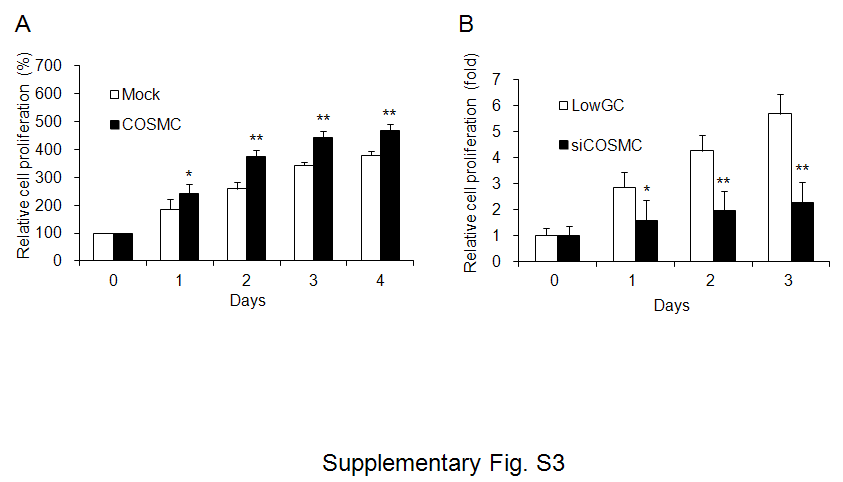

Supplement: Figure S3 — Effects of COSMC on cell proliferation. (A) COSMC overexpression increased cell proliferation of HUVEC cells. Cell proliferation was analyzed by MTT assays. (B) COSMC knockdown inhibited cell proliferation. Results are presented as means ± SD from three independent experiments. *P<0.05 and **P<0.01, compared with control. (TIF) [file pone.0056211.s003.tif]
